# Acceptability of Artificial Intelligence in Poultry Processing and Classification Efficiencies of Different Classification Models in the Categorisation of Breast Fillet Myopathies

**DOI:** 10.3389/fphys.2021.712649

**Published:** 2021-09-22

**Authors:** Aftab Siddique, Samira Shirzaei, Alice E. Smith, Jaroslav Valenta, Laura J. Garner, Amit Morey

**Affiliations:** ^1^Department of Poultry Science, Auburn University, Auburn, AL, United States; ^2^Department of Industrial and Systems Engineering and Department of Computer Science and Software Engineering, Auburn University, Auburn, AL, United States; ^3^Department of Animal Science, Czech University of Life Sciences, Prague, Czechia

**Keywords:** support vector machines, backpropagation neural networking, woody breast, meat myopathies, spaghetti meat, bioelectrical impedance analysis, machine learning, artificial intelligence

## Abstract

Breast meat from modern fast-growing big birds is affected with myopathies such as woody breast (WB), white striping, and spaghetti meat (SM). The detection and separation of the myopathy-affected meat can be carried out at processing plants using technologies such as bioelectrical impedance analysis (BIA). However, BIA raw data from myopathy-affected breast meat are extremely complicated, especially because of the overlap of these myopathies in individual breast fillets and the human error associated with the assignment of fillet categories. Previous research has shown that traditional statistical techniques such as ANOVA and regression, among others, are insufficient in categorising fillets affected with myopathies by BIA. Therefore, more complex data analysis tools can be used, such as support vector machines (SVMs) and backpropagation neural networks (BPNNs), to classify raw poultry breast myopathies using their BIA patterns, such that the technology can be beneficial for the poultry industry in detecting myopathies. Freshly deboned (3–3.5 h post slaughter) breast fillets (*n* = 100 × 3 flocks) were analysed by hand palpation for WB (0-normal; 1-mild; 2-moderate; 3-Severe) and SM (presence and absence) categorisation. BIA data (resistance and reactance) were collected on each breast fillet; the algorithm of the equipment calculated protein and fat index. The data were analysed by linear discriminant analysis (LDA), and with SVM and BPNN with 70::30: training::test data set. Compared with the LDA analysis, SVM separated WB with a higher accuracy of 71.04% for normal (data for normal and mild merged), 59.99% for moderate, and 81.48% for severe WB. Compared with SVM, the BPNN training model accurately (100%) separated normal WB fillets with and without SM, demonstrating the ability of BIA to detect SM. Supervised learning algorithms, such as SVM and BPNN, can be combined with BIA and successfully implemented in poultry processing to detect breast fillet myopathies.

## Introduction

**Figure 1:**
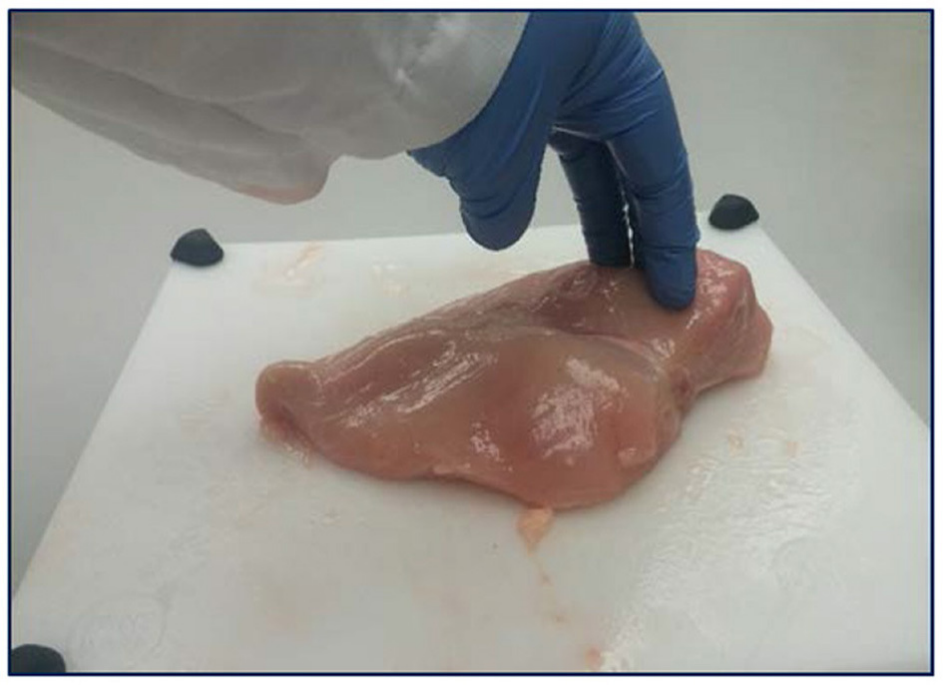
Hand-palpation method for identifying the severity of woody breast myopathy in breast fillets.

Globally, consumers are choosing meat and meat products for their higher nutritional value, especially protein (Heinz and Hautzinger, 2009). There has been a drastic increase in the consumption of these products worldwide in the last couple of decades. In developing countries, the per capita consumption of poultry has increased from 1.2 in the 1960s to 10.5 kg in the 2000s and will reach up to 14 kg by 2030 (FAO, 2003). In the United States, more than nine billion broilers were raised in 2018, with a total live weight of 27.1 billion kilogrammes; and in 2020, the per capita consumption of chicken was 44.23 kg (National Chicken Council, 2020). Chicken is a popular consumer choice because of various physicochemical and sensorial attributes such as texture, colour, and flavour (Petracci et al., 2013). To supply the increasing demand for breast meat, breeders have increased the growth rate of the birds through genetics, in turn increasing total carcass yield (Petracci and Cavani, 2012). Markets are continuously changing because of the preference and demands of consumers, which are presently driving the market toward cut-up chicken parts and further processed products. Fast-growing chickens with increased breast meat yield have developed breast muscle myopathies, leading to meat quality defects, such as woody breast (WB). In the past 10 years, WB has been more prominently found in heavier birds (Zampiga et al., 2020). Woody breast-affected fillets are characterised by an intricate and dull appearance (Sihvo et al., 2014; Kuttappan et al., 2017), and tough texture due to collagen deposition (Soglia et al., 2016). These breast myopathies also affect meat quality parameters such as pH, colour, water holding capacity (WHC), proximate composition, cook loss, and texture, which ultimately influence the quality of further processed products (Kuttappan et al., 2012). Because of lower meat quality, WB meat is sorted out in processing plants by manual hand-palpation (Figure 1) and different grading scales based on the level of severity (Table 1). However, this method is unreliable and subjective, leading to potential misclassification of the breast meat (Morey et al., 2020). By setting specific standards to accurately separate WB fillets, poultry processors will be able to reduce fillet misclassification and, ultimately, losses related to it.

**Table 1:** Different subjective scales used for the classification of woody breast meat.

**Woody breast subjective classification scale^a^**	**Condition**	**Description**
2 point scale	Normal	No toughness or hardness
	Severe	Tough fillets
3 point scale	Normal	No toughness or hardness
	Moderate	Medium toughness up to 50%
	Severe	More than 50% toughness
4 point scale	Normal	No toughness or hardness
	Mild	Hardness at cranial region
	Moderate	Fillets extremely hard and rigid through from cranial region of caudal tip fillets that were hard throughout but flexible in mid-to caudal region
	Severe	More than 50% of fillet area is woody

a *2-point scale (Sihvo et al., 2014), 3-point scale (Sihvo et al., 2014), and 4-point scale (Tijare et al., 2016)*.

We investigated bioelectrical impedance analysis as a potential objective method to detect woody breast-affected fillets. Under ideal conditions, the resistance of a conducting material is directly proportional to its length (R α L) and inversely proportional to its cross-sectional area (R α 1/A) but can be affected by the shape, size, thickness, and composition of different matrixes (Kyle et al., 2004). The electrical conductivity of a conductor depends on cell physiological and biochemical composition (the amount of water with dissolved electrolytes, intracellular fluid, extra-cellular fluid, moisture, and protein content) and on the applied signal frequency (Bera, 2014). BIA technology has been used in many species to measure physical composition and properties such as body water content and fat content. Nyboer et al. (1950) and Hoffer et al. (1969) introduced the four-electrode, whole body, and bioelectrical impedance methods in clinical studies for the measurement of bodily fluid from hand to foot. Since its inception, the use of BIA has expanded beyond clinical studies. In the food sector, BIA parameters can be calibrated to specific species and have been used in fish for the rapid detection of proximate composition and the pre-harvest condition of fish (Cox et al., 2011). The ability of meat to conduct electricity can be potentially used to detect meat myopathies such as WB and SM. WB presents itself with increased proliferation of collagen (non-conducting material), affecting resistance, while changes in intra- and extra-cellular water (conducting material) in the meat matrix alter the reactance (Kyle et al., 2004; Sihvo et al., 2014; Velleman, 2015; Soglia et al., 2016; Morey et al., 2020). Morey et al. (2020) successfully demonstrated the ability of bioelectric impedance analysis to differentiate between varying levels of WB as well as SM. The researchers attributed the differences in the electrical properties to the accumulation of collagen and increase in extra-myofibrillar water (Kennedy-Smith et al., 2017; Tasoniero et al., 2017). Contrary to WB, Morey et al. (2020) reported that loose muscle fibres acted as insulators and increased resistance readings. Morey et al. (2020) have successfully demonstrated the ability of BIA to differentiate WB and SM at different severity levels as an alternative to hand palpation to reduce human error. However, various classification algorithms should be used to further increase the accuracy of the BIA technology.

Classification accuracies of bioelectrical impedance analysis data can be improved through the use of modern data analytics techniques such as machine learning (ML), which includes data mining, artificial neural networks (ANNs), deep learning (DL), and artificial intelligence (AI) (Tufféry, 2011). ML is a complex field with a wide range of frameworks, concepts, approaches, or a combination of these methods; it is commonly used in the manufacturing sector for process optimization, tracking, and management applications in production and predictive maintenance (Wiendahl and Scholtissek, 1994; Gardner and Bicker, 2000; Alpaydin, 2010). These techniques have been widely applied to enhance quality control in production processes (Apte et al., 1993), particularly in complex production processes where predicting causes of problems is challenging (Kusiak, 2006). Over the last few decades, automated product inspection systems incorporating ML have been used in a wide variety of food industries such as potato and apple (Tao et al., 1995), oil palm fruit (Abdullah et al., 2002), rice and grains (Carter et al., 2005), beef fat (Chen et al., 2010), and colour in bakery applications (Nashat et al., 2011).

The use of machine learning models has increased in recent years because of circumstances such as the availability of complex data with little accountability (Smola and Vishwanathan, 2008) and will become more critical in the future. Although several ML algorithms are available, such as ANNs, support vector machines (SVMs), and distributed hierarchical decision trees, their ability to deal with large data sets varies significantly (Monostori, 2003; Bar-Or et al., 2005; Do et al., 2010). In the production sector, only specific ML algorithms are capable of handling high-dimensional datasets, and having the ability to deal with high dimensionality is considered a benefit of using ML in the processing industry. One of the main benefits of ML algorithms is finding previously unknown (hidden) information and recognising its associations in large datasets. The available information criteria can depend mainly on the characteristics of an ML algorithm (supervised/unsupervised or reinforcement learning, RL). Nevertheless, the general process of the ML method for producing outcomes in a production environment has been proven conclusively (Filipič and Junkar, 2000; Guo et al., 2008; Alpaydin, 2010; Kala, 2012). The use of the BIA method in poultry processing provides complex data with high dimensionality, which can be used to train SVM algorithms for the classification of WB (based on severity) and SM fillets. Support vector machines (SVMs), with a kernel-based procedure, has emerged in machine learning as one strategy for sample classification (Pardo and Sberveglieri, 2005). The implication of SVMs in machine learning as a supervised learning technique provides good generalisation ability and more minor overfitting tendencies. Using kernel functions in SVMs makes original input values linearly separable in a higher dimensional space. Moreover, SVMs can simultaneously reduce estimation errors and model dimensions (Singh et al., 2011). The main objective of this research was to determine the accuracy of linear discriminant analysis (LDA), SVMs, and backpropagation neural networks (BPNNs) to classify WB and SM using multi-dimensional BIA data. The LDA, SVM, and BPNN methods are discussed in detail, their accuracies were compared, and reasons for the differences in the classification accuracies are discussed.

Poultry researchers and the industry collect enormous amounts of data on a regular basis but use simpler statistical methods to derive meaning from the data. Through the presented research, we envision to introduce the poultry research community to several data analytics techniques to analyze complex datasets.

## Materials and Methods

### Data Collection

**Figure 2:**
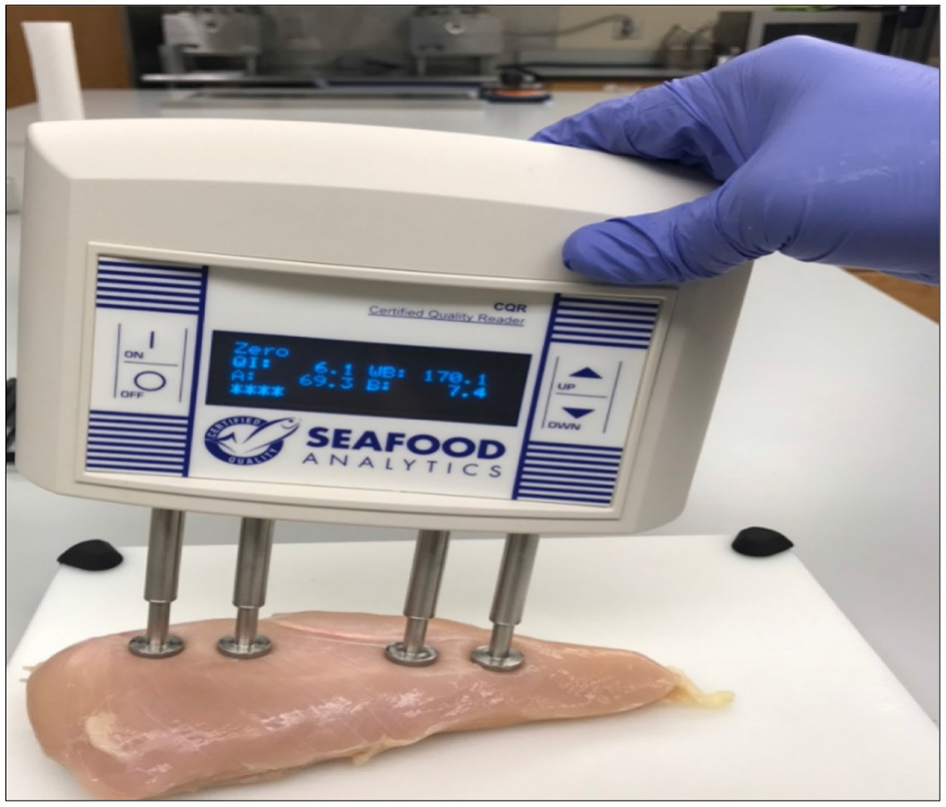
Hand-held bioelectrical impedance device to measure the severity level of fillets.

Freshly deboned breast fillets from 56-day old broilers (Ross 708) were analysed in a federally inspected commercial poultry processing facility after deboning. The breast fillets (*n* = 300, 3 replications or flocks) were randomly selected from the processing line 3 to 3.5 h post slaughter. The deboned breast fillets were analysed for WB incidence through hand palpation by an experienced team member (Figure 1). The breast fillets were classified into normal, mild (for data analysis mild was grouped with normal), moderate, and severe WB fillets (Tijare et al., 2016), and SM presence was evaluated by observing the turgor in the cranial-ventral portion of the breast fillets, with a decrease in turgor indicating the presence of SM and increase in turgor representing the absence of SM. The collected chicken breast fillets from the processing line were subjected to BIA by utilising a hand-held CQ Reader (Figure 2; Seafood Analytics, Clinton Town, MI, United States) (Morey et al., 2020), equipped with four spring-loaded electrodes (RJL Systems, Detroit, MI, United States). All the four electrodes were placed to make contact with the ventral surface of the breast fillets. Once the electrodes were in contact with the breast fillets, the circuit was complete and linked. Then, the device measured the data for resistance, reactance, fat index, and protein index, and the stored data were downloaded for analysis later (Seafood Analytics Certified Quality Reader, Version 3.0.0.3; Seafood Analytics, MI, United States). Individual weights of the fillets were also determined using a weighing balance (Ohaus Corporation, Pine Brook, NJ, United States) for the analysis and used to train the SVM and BPNN models.

### Linear Discriminant Analysis

**Figure 3:**
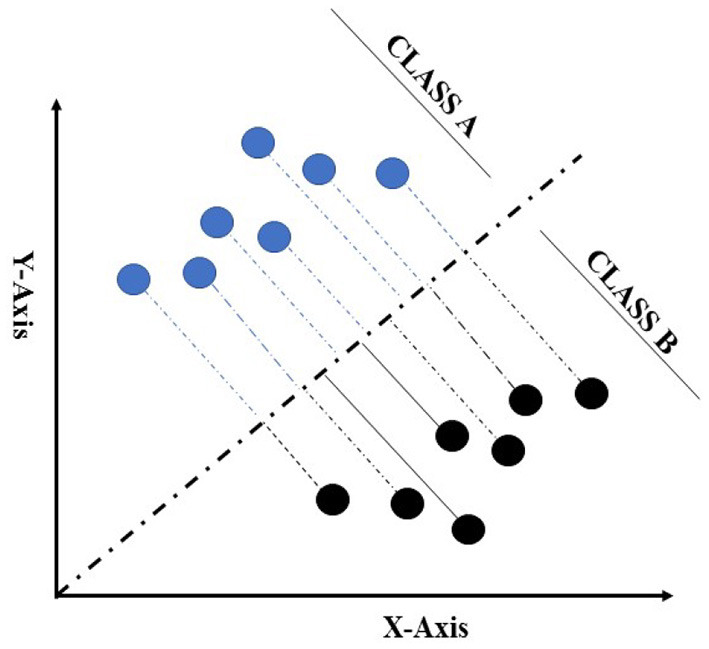
Representation of two-class data in dimensional space for linear discriminant analysis (LDA) to maximise the classifiable data on the hyper-plane. This Figure adapted from Fisher (1936).

Linear discriminant analysis is one of the conventional data mining algorithms used in supervised and unsupervised learning contemplated by Fisher (1936) for resolving the issue related to flower classification (Xanthopoulos et al., 2013). The LDA model is used to project an imaginary hyper-plane that minimises the interclass variance and maximises the distance between class means. Additionally, it produces a transformation in the data that is discriminative in some data cases (Fukunaga, 2013). LDA is more appropriate for data where unequal within-class frequencies are given, and its classification performances have been randomly examined on generated test data. This approach maximises the ratio of between-class variance to within-class variance with maximum separability. Data sets used in LDA analysis can be transformed, and related test vectors can be classified in the imaginary hyper-plane by class-dependent transformation and class independent transformation (Balakrishnama and Ganapathiraju, 1998). The class-dependent transformation approach maximises the ratio of between-class variance to within-class variance. This kind of class transformation helps in maximising class separability (Tharwat et al., 2017). The main objective for implementing LDA is to create a subspace of lower-dimensional data points compared with the sample data set, in which the original data points from the data set can be easily separable (Figure 3; Fisher, 1936). The use of LDA provides a solution that can be implemented in a generalised eigenvalue system, which provides huge and fast data optimization. The original LDA algorithm was used to solve binary classification taxonomic problems; however, Rao (1948) had also proposed multi-class generalisations. In this study, both class classification and multi-class case classification derivation were provided to better understand the concept from the simple two-class case (Xanthopoulos et al., 2013).

Let “a1,., a_p_ ∈ R^m^” be a set of “q” data sets related to the two separate classes, A and B. For each class defined, sample means are


(1.1) $$\begin{array}{l} a_{\text{A}}=\text{I}{\text{N}}_{\text{A}}\sum \text{a}\in {\text{A}}_{\text{a},},\;\bar{\text{a}}\text{B}=1{\text{N}}_{\text{B}}\sum \text{a}\in {\text{B}}_{\text{a}.} \end{array}$$

N_A_, N_B_ is the total number of samples in data sets A and B. Scatter matrices for the data set by the equation


(1.2) $$\begin{array}{r} {\text{S}}_{\text{A}}=\sum \text{a}\in \text{A}\left(\text{a}-{\bar{\text{a}}}_{\text{A}}\right){\left(\text{a}-{\bar{\text{a}}}_{\text{A}}\right)}^{\text{T}},\\ {\text{S}}_{\text{B}}=\sum \text{aϵB}\left(\text{a}-{\bar{\text{a}}}_{\text{B}}\right){\left(\text{a}-{\bar{\text{a}}}_{\text{B}}\right)}^{\text{T}} \end{array}$$

Each of the matrices mentioned above is used for the imaginary hyper-plane, which is defined by the vector (φ), and the variance for the calculation is minimal and can be explained by the equation


(1.3) $$\begin{array}{r} Min\;\varphi \left(\varphi^{T}s_{A}\varphi +\varphi^{T}s_{B}\varphi \right)=min\;\varphi \varphi^{T}\left(s_{A}+s_{B}\right)\\ \varphi =min\varphi \varphi^{T}s_{\phi } \end{array}$$

where S = S_A_ + S_B_ by definition and from equations 1.2, the scatter matrix for supposed two matrixes for the two classes are


(1.4) $$\begin{array}{l} s_{AB}=\left({\bar{a}}_{A}-{\bar{a}}_{B}\right){\left({\bar{a}}_{A}-{\bar{a}}_{B}\right)}^{T}. \end{array}$$

LDA is based on Fisher's projection hyper-plane, i.e., maximizing the distance between the means and minimizing the variance of each considered class that can be mathematically described by Fisher's criterion equation as:


(1.5) $$\begin{array}{l} Max\varphi J\left(\varphi \right)=max\;\varphi \varphi^{T}s_{AB\varphi }\varphi^{T}S_{\varphi }. \end{array}$$

There could be several solutions for the optimization-related problem with the same function value. For a solution ϕ,* all the vectors c·φ* will give the same value, and considering no loss in generality, we select only one best possible solution by substituting the denominator with an equality constraint. Then, the problem becomes


(1.6a) $$\begin{array}{l} Max\;\varphi \varphi^{T}S_{AB}\varphi \end{array}$$

(1.6b) $$\begin{array}{l} s.t\cdot \varphi^{T}s_{\varphi }=1 \end{array}$$

The Lagrangian mechanism associated with this problem is


(1.7) $$\begin{array}{l} LLDA\left(a_{,}\text{λ}\right)=\varphi^{T}S_{AB}\varphi -\text{λ}\left(\varphi TS_{\varphi }-1\right) \end{array}$$

where λ is the LaGrange multiplier associated with the equation 1.6b. Since S_AB_ is positive and the nature of the problem is convex, the global minimum will be at the point for which


(1.8) $$\begin{array}{l} \partial LLDA\left(x_{,}\text{λ}\right)/\partial x=0\Leftrightarrow S_{AB}\varphi -\text{λ}S_{\varphi }=0. \end{array}$$

The optimal φ obtained as the eigenvector that corresponds to the smallest value for the generalised eigensystem:


(1.9) $$\begin{array}{l} S_{AB}\varphi =\text{λ}S_{\varphi }. \end{array}$$

Multi-class LDA is only the extension of the two-class classification problem. Given x classes, the matrices will be redefined, and the intra-class matrix becomes


(1.10) $$\begin{array}{l} S=S_{1}+S_{2}+\ldots .S_{n,} \end{array}$$

while the inter-class scatter matrix is annotated by


(1.11) $$\begin{array}{l} S_{1,\ldots }n=n\sum i=1pi\left({\bar{a}}_{i}-\bar{a}\right){\left({\bar{a}}_{i}-\bar{a}\right)}^{T}, \end{array}$$

where the number of samples (p_i_) in the ith class, a_i_ is the mean, and a is mean vector given in equation


$$\begin{array}{l} \bar{a}=1pn\sum i=1pi\;¯ai. \end{array}$$

The linear transformation φ can be achieved by solving the above equation:


$$\begin{array}{l} S1,...,n\;\varphi \;=\text{λ}S\varphi \end{array}$$

to achieve a better classification by projection of the hyper-plane. Once the transformation ϕ is achieved, the class of a new point “y” is determined by


(1.12) $$\begin{array}{l} class\left(y\right)=arg\;minn\left\{d\left(y\varphi ,\;¯an\varphi \right)\right\} \end{array}$$

where a_n_ is the centroid of nth class. The calculation reflects that all the centroids of the classes were defined first and that the unknown points on the subspace were defined by φ and the closest class concerning D.

### Support Vector Machines

**Figure 4:**
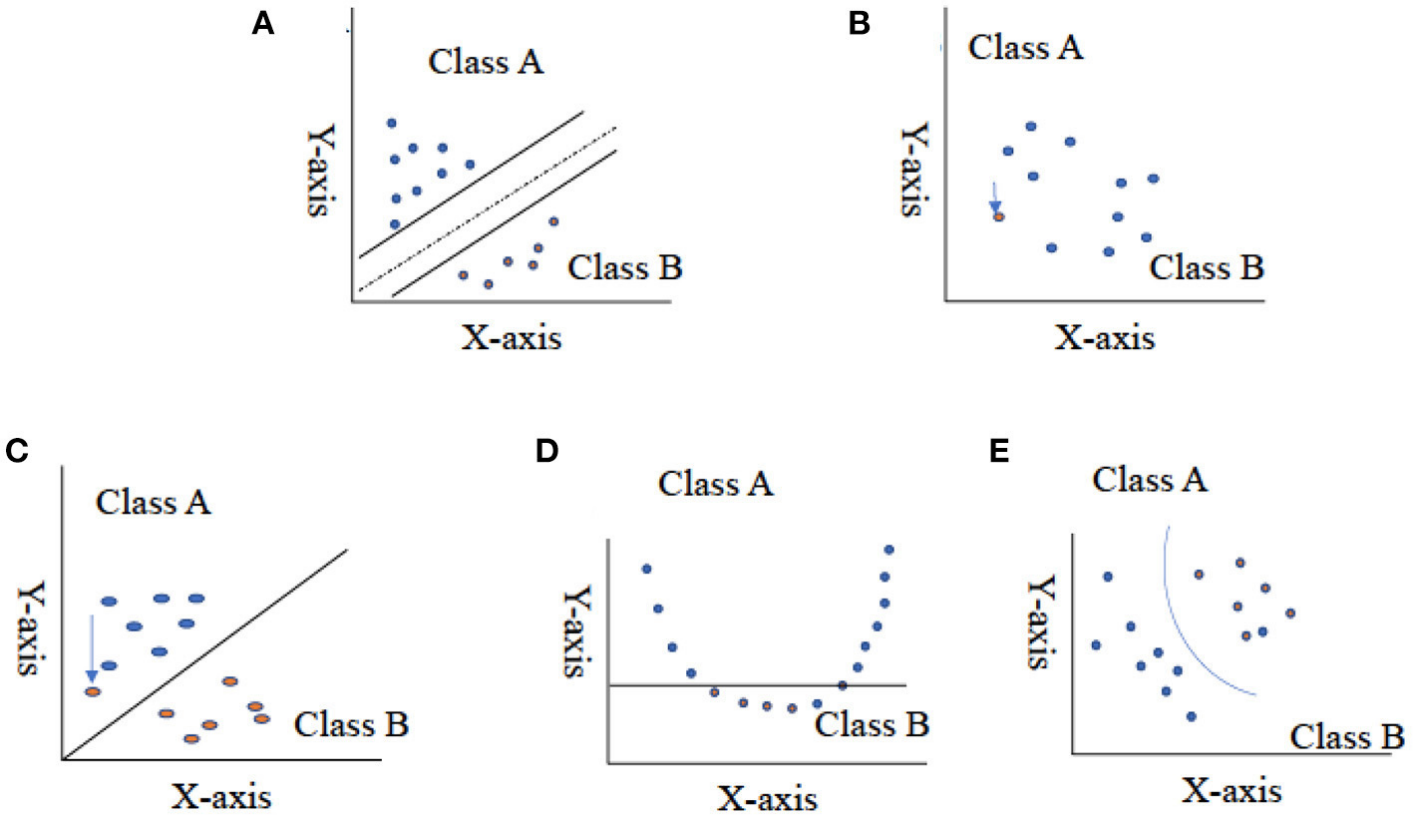
Representation of two-class data using hyper-plane for support vector machine. **(A)** Represents split data set in half, **(B)** represents the two-close value, **(C)** represents the outlier value as the solution for the figure **(B)**, **(D)** represents non-linear data set, and **(E)** represents the use of kernel function and change in dimensionality of data. This Figure adapted from Noble (2006).

Vapnik (2013) first contemplated the support vector machine method in 1995, and recently it has enticed an enormous level of endeavour in the machine learning applications community. Several studies have mentioned that the SVM method has immense performance in classification accuracy compared with other data classification algorithm methods (Maji et al., 2008; Shao and Lunetta, 2012; Vijayarani et al., 2015). SVM generates a line between two or more classes known as hyper-planes for data set classification. Input data Q that can fall on either side of the hyper-plane (QT•W–b) > 0 are labelled as +1, and those that fall on the other side, (QT•W–b) < 0, are labelled as −1 (Figure 4A; Lee and To, 2010); let {Qi, yi} ∈ R^n^ be training data set, y_i_∈{1, −1}, i = 1, 2,…,n.

There exits hyper-plane,


(2) $$\begin{array}{l} P\;=\;\left\{Q\in \;Rn/QT•W+\;b\;=\;0\right\} \end{array}$$

The equation for the training data set can be written as


(2.1) $$\begin{array}{r} QiT\;W\;+\;b\;\geq 1,\;yi\;=1\\ QiT\;W\;+\;b\;\geq -1,\;yi\;=-1 \end{array}$$

The above-mentioned equations can be written as


$$\begin{array}{l} yi\;\left(QiT\;W\;+\;b\;-1\;\right)\;\geq \;0 \end{array}$$

Another definition for the hyper-plane considering P^−^ and P^+^, let {Qi, yi} ∈ *R*^*n*^ be training data set, y iÎ {1, −1}, i = 1,2,…,n,


(2.2) $$\begin{array}{r} P+=\left\{Q\;\in \;Rn\;/QTi\;W\;+\;b\;=1\right\}\\ P-=\left\{Q\;\in \;Rn\;/QTi\;W\;+\;b\;=-1\right\} \end{array}$$

The optimization mentioned above is a form non-convex optimization problem that relies on the absolute value of |W| and is difficult to solve than convex optimization problems. The equation for the absolute value W can be replaced using 1/ 2 | **|**W| |^2^ without having any change in the final solution; so, the representation of the SVM-related problem in quadratic programming (QP) form is as follows (Osuna et al., 1997):


(2.3) $$\begin{array}{r} Min\;1/\;2\;|\;|W|\;|2,\\ st.yi\left(Q_{i}^{T}W+b\right)-1\geq 0,1\leq i\leq n \end{array}$$

After solving the SVM optimization problem using Lagrange multipliers (a_i_), the Wolfe dual of the optimization problem was achieved (Craven, 1989):


(2.4) $$\begin{array}{l} L\left(w,b\right)=\frac{1}{2}||w{||}^{2}-\sum_{i=1}^{n}aiyi\left[\left(Q_{i}^{T}W+b\right)-1\right]. \end{array}$$

After solving for the value for W and b,


(2.5) $$\begin{array}{l} \frac{\;\partial L\left(w,b\right)}{\partial w}=0,\;\frac{\partial L\left(w,b\right)}{\partial b}=0 \end{array}$$

the solution in Equation (2.5) is the following condition:


(2.6) $$\begin{array}{r} w=\sum_{i=1}^{n}\alpha_{i}yiQi\\ \sum_{i=1}^{n}\alpha_{i}yi=0 \end{array}$$

putting the value of 2.6 into equation 2.4, we get the dual form of SVM,


(2.7) $$\begin{array}{r} W\left(a\right)=\sum_{i=1}\alpha_{i}-\left(\sum_{i=1}^{n}\alpha_{i}\alpha_{j}y_{i}y_{j}\left(Q_{i}\cdot Q_{j}\right)\right)/2,\\ st.\sum_{i=1}^{n}\alpha_{i}y_{i}\left(Q_{i}\cdot Q_{j}\right)=0\\ 0\leq ai\leq c,\;i=1,2,.\;.\;.\;.\;.\;n \end{array}$$

The number of variables in the Equation derived is equivalent to the total number of data cases (n). The training set data with a_i_ > 0 represents the position of support vectors for the classification, and *Qi p*+ *or Q*_*i*_*p–*.

The equation for hyper-plane decision can be written as Pontil and Verri (1998)


(2.8) $$\begin{array}{l} f\left(x\right)=\pm \left(\sum_{i=1}^{n}\alpha_{i}^{*}yi\left(q\cdot q_{i}\right)-b^{*}\right) \end{array}$$

where q is the unknown input data that need to be classified. The SVM method has been employed in a considerable range of real-world problems associated with different fields of automation, forensics, biotechnology, agriculture statistics, and is now being used in food sciences for the classification of bakery products, fresh produce, and meat product classifications (Liu et al., 2013; Chen and Zhang, 2014; Asmara et al., 2017; Chen et al., 2017; Arsalane et al., 2018). It has been proven that SVMs are persistently most appropriate for diverse supervised learning methods. Despite this, the performance of SVMs is very receptive to the cost parameter, and kernel frameworks are set. As a result, research industries want to conduct ample cross-validation to determine the most influential parameter setting (Durgesh and Lekha, 2010).

### Backpropagation Neural Networking

According to Lippmann (1987), there were no practical algorithms available for interconnecting weight values to achieve an overall minimum training error in multilayer networks. Rumelhart et al. (1986) proposed a generalised rule for backpropagation neural networking, an iterative, gradient descent training procedure. The input data, in the form of vector, are a pattern to be learned, and the desired output is in the form of a vector produced by the network upon recall of the input training pattern (Paola and Schowengerdt, 1995). The main goal of the training is to minimise the overall error between the test set data and training set data outputs of the network (Paola and Schowengerdt, 1995). BPNN is also recognized as multilayer perceptrons, one of the multiple layers forward neural networks. A BPNN comprises one input layer, one or more hidden layers, and one output layer (Bharathi and Subashini, 2011; Liu et al., 2013). Consideration of distinct factors plays a fundamental role when developing a BPNN that consists of the structure of network, initialisation, and switch functions in each hidden and output layer, the training way and algorithm, the learning rate, the error-goal (ε), and preprocessed input data. BPNN has some advantages, such as easy architecture, ease of assembling the mannequin, and fast calculation speed. However, BPNNs have some issues, such as (i) possible to contain in local extremum, (ii) poor generalisation ability, (iii) lack of strict format packages with a theoretical foundation, and (iv) challenging to manage the learning and training method (Yao, 1999).

**Figure 5:**
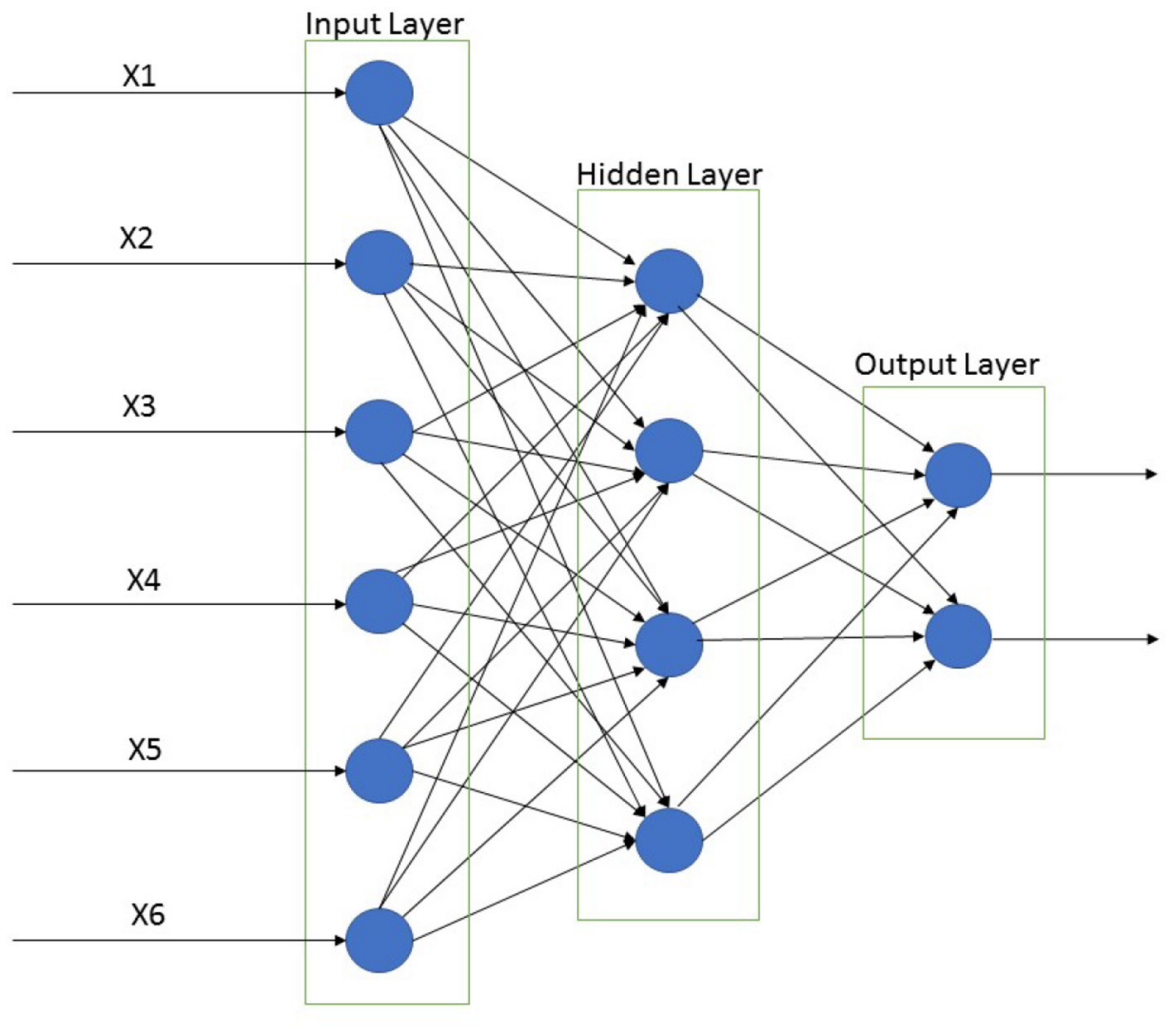
Back propagation neural network classification for input, hidden, and output layer. This Figure was adapted from Rumelhart et al. (1986).

In spite of these problems, backpropagation neural networks have been successfully implemented in a range of fields. Users have applied their experiences and prior knowledge during the design of a BPNN to overcome these problems (Liu et al., 2013). A supervised BPNN learning algorithm consists of an input layer, one or more hidden layers, an output layer, and the nodes of the hidden layers primarily affect the classification efficiency of the neural network (Figure 5). Parameters that are required to be defined by the users are learning rate (0 < η < 1) and momentum (0 < η < 1).

BPNN program training procedure (Lee and To, 2010; Yang et al., 2011):

1. Design and input for network.
2. Normalise the initial input weights W and threshold values (θ).
3. Define the training and testing data set and input the training matrix X and output matrix Y.
4. Estimate the output vector of each neural synaptic unit.
  (a). Evaluate the output vector (Z) for the hidden layer:
    (3.1) $$\begin{array}{l} net_{k}=\Sigma w_{ik}x_{i}-\theta_{k,} \end{array}$$
    (3.2) $$\begin{array}{l} Zk\;=\;f\;\left(net\;k\right) \end{array}$$
    (3.3) $$\begin{array}{l} net_{j}=\Sigma w_{kj}Z_{i}-\theta_{j,} \end{array}$$
    (3.4) $$\begin{array}{l} Y_{\text{j }}=\;f\left({\text{net}}_{\text{j}}\right)\left(3.4\right) \end{array}$$
  (b). The root of the mean square:
    (3.5) $$\begin{array}{l} RMS=\sqrt{\frac{\sum {\left(y_{j}-T_{j}\right)}^{2}}{n}} \end{array}$$
5. Estimate distance δ for the output layer and hidden layer from Equations (3.6, 3.7):
  (3.6) $$\begin{array}{l} \delta_{j}=\left(T_{j}-y_{i}\right)-f\left(net_{j}\right) \end{array}$$
  (3.7) $$\begin{array}{l} \delta_{k}=\left(\sum_{j}\delta_{j}w_{kj}\right)-f^{{\;}^{\prime }\left(net_{j}\right)} \end{array}$$
6. Evaluate modifications for initial weights (W) and distance (δ) (η is the learning rate, α is the momentum) for both output layer (Equations 3.8, 3.9) and hidden layer (Equations 3.10, 3.11):
  (3.8) $$\begin{array}{l} \Delta w_{kj\left(n\right)}=\eta \delta_{j}z_{k}+\alpha \Delta w_{Kj}\left(n-1\right) \end{array}$$
  (3.9) $$\begin{array}{l} \Delta \theta_{j}\left(n\right)=-\eta \delta_{j}+\alpha \Delta \theta_{j}\left(n-1\right) \end{array}$$
  (3.10) $$\begin{array}{l} \Delta w_{ik\left(n\right)}=\eta \delta_{j}X_{i}+\alpha \Delta w_{ik}\left(n-1\right) \end{array}$$
  (3.11) $$\begin{array}{l} \Delta \theta_{k}\left(n\right)=-\eta \delta_{k}+\alpha \Delta \theta_{k}\left(n-1\right) \end{array}$$
7. Redefine initial weight (W) and the threshold value (θ), redefine W and θ of the output and hidden layer:
  (3.12) $$\begin{array}{l} w_{kj}\left(p\right)=w_{kj}\left(P-1\right)+\Delta w_{k_{j}} \end{array}$$
  (3.13) $$\begin{array}{l} \theta_{j}\left(p\right)=\theta_{j}\left(p-1\right)+\Delta \theta_{j} \end{array}$$
  (3.14) $$\begin{array}{l} w_{ik}\left(p\right)=w_{ik}\left(P-1\right)+\Delta w_{ik} \end{array}$$
  (3.15) $$\begin{array}{l} \theta_{k}\left(p\right)=\theta_{k}\left(p-1\right)+\Delta \theta_{k} \end{array}$$

After modifying output and hidden layer, the steps will be renewed, and steps 3–7 will be repeated until converge.

BPNN program-testing process (Lee and To, 2010; Yang et al., 2011):

1. Input parameters related to the network.
2. Input the initial weights (W) and the threshold value (θ).
3. Unknown data entry for data matrix X.
4. Evaluate output vector (Z) for the output and hidden layer:

(3.16) $$\begin{array}{l} net_{k}=\Sigma W_{ik}x_{i}-\theta_{k} \end{array}$$

(3.17) $$\begin{array}{l} z_{k}=f\left(nt_{k}\right) \end{array}$$

(3.18) $$\begin{array}{l} nt_{j}=\Sigma w_{k\dot{j}}z_{i}-\theta_{j} \end{array}$$

(3.19) $$\begin{array}{l} Y_{j}=f\left(nt_{j}\right) \end{array}$$

### Statistical Analysis

All the parameters (resistance, reactance, fat index, protein index, and fillet weights) were analysed by one-way analysis of variance with Tukey's honestly significant difference (HSD) (*p* < 0.05) to determine significant differences among the levels of myopathy severity. The data were further analysed using three classification methods, linear discriminant analysis (LDA) (SAS, Version 9.4), support vector machines (SVMs), and backpropagation neural networking (BPNN). For each method, the data were analysed using three different scenarios: (1) all data (WB scores, fillet weight, resistance, reactance, protein index, and fat index); (2) without fillet weights (WB scores, resistance, reactance, protein index, and fat index); and (3) without fat and protein index (WB scores, fillet weight, resistance and reactance). For the SVM and BPNN analysis of collected data, R software (Version 4.0.0, Arbour Day) was used by using the caret package in the analysis to classify various chicken breast fillet myopathies. The data sets collected for the different conditions were divided into 70::30 training set and testing set. The caret package algorithm calculated the best-suited tuning parameter or value of cost (C) for both the training and testing data sets. A seed value was set for 3,000 for the SVM analysis. For BPNN the classification of fillets, the Neural net and BBmisc packages were used to classify the collected data sets (WB and SM), and the data sets were divided into 70::30 training and testing data sets. Low learning rate (0.01), the threshold value (0.01), number of maximum steps (10,000), and four hidden layers were used in the BPNN classification algorithm for the analysis.

## Results

The differences in various parameters among the levels of woody breast and spaghetti meat severity are shown in Tables 2, 3, respectively. As shown in Table 2, there are significant differences (*p* < 0.05) in the resistance, reactance, and fillet weights among the WB categories. There were no significant differences (*p* > 0.05) in the fat and protein index between WB severity levels (Table 2). On the contrary, there are no significant differences in the parameters between breast fillets with and without SM (Table 3). Classification efficiencies of the fillets are greatly affected by the analysis of all the data, without fillet weights and without fat and protein index, indicating the significance of variables in classification accuracy (Table 2). The LDA, SVM, and BPNN analyses showed that when the fillet weights were removed from the dataset, classification accuracy (testing %) was reduced by as high as 20% (Table 2). The LDA analysis without fillet weight data set showed less classification efficiency in the testing data set (normal = 43.8%, moderate = 17.2%, and severe = 50.0%) compared with the training data set (normal= 62.1%, moderate = 32.2%, and severe = 64%), indicating that the training model was not efficient in classification at the testing stage. Similarly, the SVM analysis showed low classification efficiency for the testing set (normal = 56.52%, moderate = 49.77%, and severe = 64.63%). The removal of fat and protein index data from the analysis showed that the LDA analysis for normal (training::testing = 61.7::75.6) and moderate fillets (training::testing = 31.3::33.33) improved as compared with the analysis without the fillet weight data set and all the data sets (including resistance, reactance, protein index, fat index, and fillet weights). The lower efficiency may be due to the misclassification of fillets due to fat and protein index value overlapping. The SVM analysis showed a higher classification pattern for normal (training::testing = 65.75::73.28) and severe breast fillets (training::testing = 72.49::81.48), similar to the overall data set. For the classification of SM fillets from the normal fillets, the SVM analysis performed better in the classification of normal fillets (training::testing = 57.06::60.00) from spaghetti meat-conditioned fillets (training::testing = 45.5::22.2) (Table 2). On the other hand, the SVM analysis for the resistance, reactance, and weight data set showed slightly improved classification efficiency for fillets with SM condition (training::testing = 50.00::52.35). BPNN did not perform well in any of the given conditions for the classification of fillets based on WB severity and SM fillets conditions.

**Table 2:** Comparison of bioelectrical impedance parameters and fillet weights among woody breast fillets with varying myopathy severity levels.

**Condition**	**No. of fillets**	**Resistance**	**Reactance**	**Fat index**	**Protein index**	**Weight of fillets**
Normal	148	71.89 ± 0.52^a^	36.93 ± 0.44^a^	13.27 ± 0.21^a^	37.71 ± 0.27^a^	455.80 ± 3.85^c^
Moderate	82	68.78 ± 0.55^b^	32.21 ± 0.49^b^	13.32 ± 0.21^a^	37.56 ± 0.27^a^	485.66 ± 3.80^b^
Severe	70	67.90 ± 0.55^b^	30.72 ± 0.50^c^	14.09 ± 0.21^a^	37.11 ± 0.27^a^	514.86 ± 3.75^a^

*Means with different superscript are significantly different from each other*.

**Table 3:** Comparison of bioelectrical impedance parameters and fillet weights among normal breast fillets with and without spaghetti meat conditions.

**Parameters**	**Spaghetti meat (Yes)**	**Spaghetti meat (No)**
Resistance	69.88 ± 0.52^a^	70.19 ± 0.52^a^
Reactance	35.63 ± 0.44^a^	34.08 ± 0.44^a^
Fat index	13.73 ± 0.21^a^	13.39 ± 0.21^a^
Protein index	37.36 ± 0.27^a^	38.05 ± 0.27^a^
Weight of fillets	473.26 ± 3.75^a^	479.24 ± 3.75^a^

*Spaghetti meat (Yes = 75; No = 225).*

a *means with same letter superscript are not significantly different*.

## Discussion

**Figure 6:**
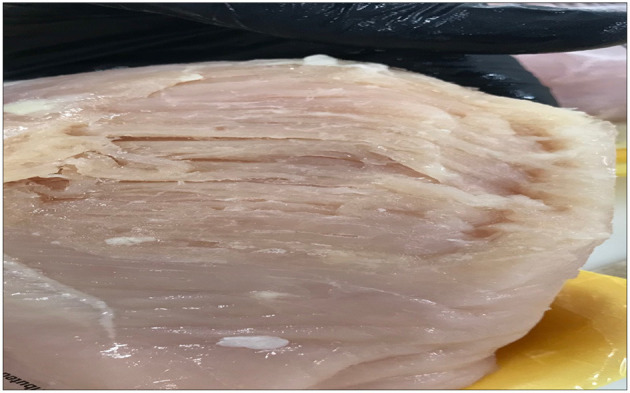
Spaghetti meat condition in chicken breast fillets.

**Figure 7:**
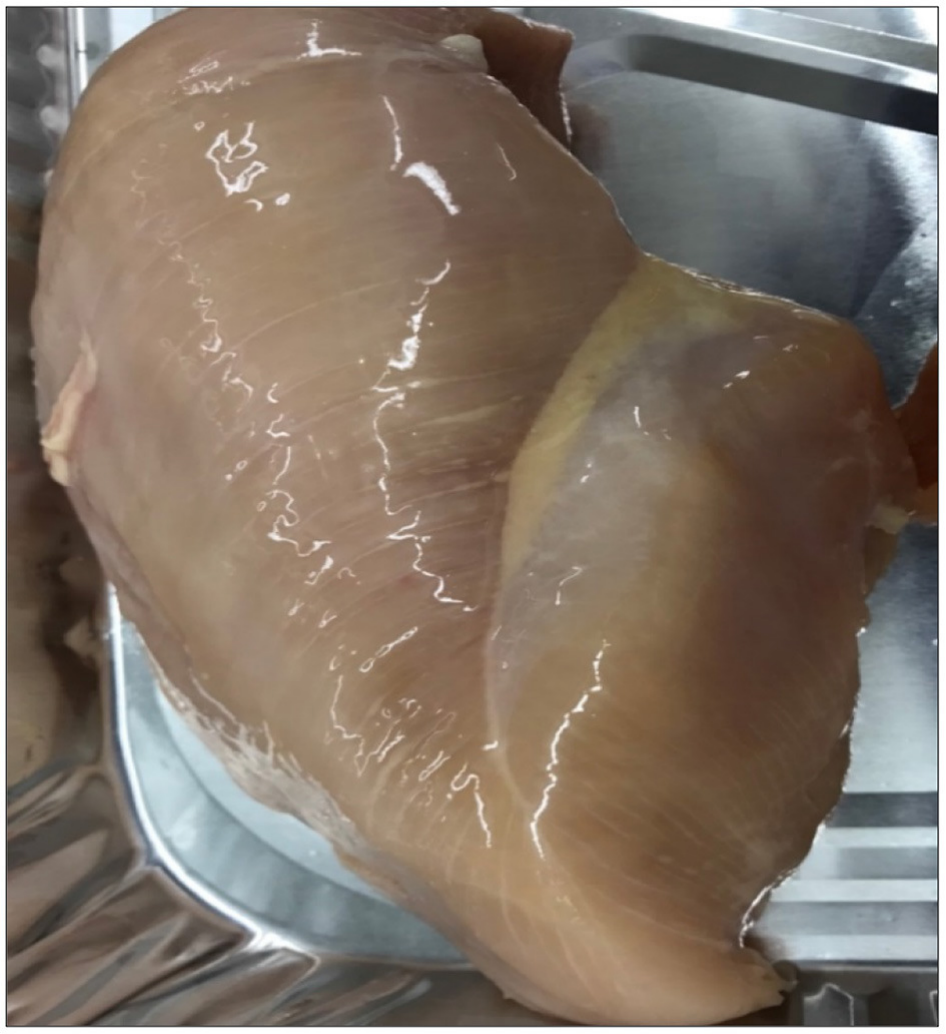
Severe woody breast fillet in the collected samples.

Using only visual and hand palpation characteristics to identify woody breast and spaghetti meat muscle myopathies poses various challenges, such as misclassifications, processing inefficiencies, and increase in labour costs, when classification is performed on a processing line. WB is found primarily in the superficial area of breast fillets and, many times, includes the visual presence of surface haemorrhages, appearance of light-yellow surface, rigid bulged fillet, and by mechanical palpability of the muscle (Figure 6; Mazzoni et al., 2015; Mudalal et al., 2015). Additionally, normal breast fillets have smaller cross-sectional areas as compared with woody breast fillets (Huang and Ahn, 2018), with higher collagen content and elevated post processing pH (Petracci et al., 2015; Chatterjee et al., 2016; Clark and Velleman, 2016; Soglia et al., 2016). SM, on the other hand, is related to immature intramuscular connective tissues in the breast meat, and it has lower muscular cohesion than the breast meat from unaffected fillets (Figure 7; Bowker and Zhuang, 2016; Radaelli et al., 2017; Sihvo et al., 2017). The thickness of connective tissues in the breast fillets showing SM decreases gradually in the endomysium and perimysium, causing different muscle fibres to deteriorate or have a mushy texture (Baldi et al., 2018). Therefore, using an assortment of already available complex data, we were able to make improvements to the classification of fillets among the WB and SM myopathies.

Significant differences were observed in the resistance and reactance among the normal, moderate, and severe woody breast fillets (Table 2), indicating changes in the muscle architecture and the intra- and extra-cellular water contents of the meat (Morey et al., 2020). It was expected to find significant differences in the fillet weights among the levels of WB severity, with severe WB fillets weighing significantly heavier than the normal fillets (*p* < 0.05). However, contrary to Morey et al. (2020), this research reports an inverse trend in resistance and reactance of normal and severe woody breast. Morey et al. (2020) reported that resistance and reactance were lower in normal meat (72.18 and 28.04 Ω, respectively), and higher in severe WB meat (78.27 and 37.54 Ω). In this study, resistance and reactance were higher in normal meat (71.89 and 36.93 Ω) and lower in severe WB meat (67.9 and 30.72 Ω). As for the normal meat with and without SM (Table 3), the observations were contrary to Morey et al. (2020). This study (Table 3) did not find significant differences in the resistance, reactance, fat index, protein index and weight of the normal fillets with and without SM.

In this study, the breast fillets were taken from water-chilled birds, which were immediately deboned on a deboning line and analysed in a plant, while in the study by Morey et al. (2020), the fillets were transported to a lab and analysed within 6 h of procurement. It would be of interest to investigate if the differences in bioelectrical properties are (1) water-retention in the normal breast fillets due to immersion chilling and (2) post deboning holding time. The findings emphasise the fact that the bioelectrical parameters were standardised by the processors prior to use.

The results obtained with training accuracy for linear discriminant analysis (70::30) classification were 72.31, 43.75, and 75% for the normal, moderate, and severe woody breast (Table 4) fillet classifications, respectively, using the bioelectrical impedance analysis and fillet weight data set (*n* = 300). The testing set was lower in accuracy than the training set, with only 52.63% normal classified, 29.41% moderate classified, and 59.09% severe WB classified (*n* = 300; Table 4). The testing data set was lower in accuracy compared with the training set data, possibly because of the low sample size and non-linearity of the data set. The non-linear data set is likely due to human error during the manual hand-palpation of the breast fillets; however, in future studies, larger data sets could be implemented to increase the accuracy of the BIA method combined with conventional algorithms. Morey et al. (2020) also performed LDA (60::40) with a BIA data set (*n* = 120) and reported 68.69–70.55% accuracy for the classification of normal fillets and 54.42–57.75% accuracy for severe WB fillets in the testing set. Wold et al. (2019) analysed a near infrared spectroscopy (NIR) data set (*n* = 102) using an LDA (50::50) classification algorithm with 100% accuracy for fillet classification in the training set and 96% accuracy in the testing set, for a rapid on-line detection method for WB myopathy in processing plants. LDA is a well-recognised technique for reducing the dimensionality of data in a dataset. However, LDA can only be used for single-label multi-class categorizations and cannot explicitly be extended to multi-label multi-class classification systems. The LDA technique is used to convert high-dimensional data into a low-dimensional data space, maximising the ratio of between-class variation to within-class variance, thereby ensuring optimal class separation (Pan et al., 2014). The LDA technique works by projecting the initial data matrix onto a lower-dimensional region. For the reduction of dimensionality, three steps are required: (i) the inter-class difference or between-class matrix is used to measure the separability across multiple categories (i.e., the distance between the means of different classes), (ii) the within-class variance, also known as the within-class matrix, is calculated as the difference between the mean and the class samples, and (iii) the creation of a lower-dimensional space that maximises between-class variance while minimising within-class variance (Mandal et al., 2009). In this research and that of Morey et al. (2020), the low performance of data collected and analysed by LDA compared with the data collected may have two key factors: small sample size and data linearity issues. Su et al. (2017) also found low performance in data sets with small sample size and non-linear data.

**Table 4:** Percentage classification efficiency for various supervised machine-learning algorithms (linear discriminant analysis, support vector machines, and back propagation neural networking) for breast fillets with woody breast^a^ and spaghetti meat^b^ in three different scenarios (all data, without fillet weights, and without fat and protein index value).

**Classification method**	**Subjective classification**	**Accuracy** **(all data)** ^**c**^		**Accuracy** **(without fillet weights)** ^**d**^		**Accuracy** **(without** **fat and protein index)** ^**e**^	
		**Training (%)**	**Testing (%)**	**Training (%)**	**Testing (%)**	**Training (%)**	**Testing (%)**
**Woody breast meat**							
Linear discriminant analysis	Normal	72.31	52.63	62.10	43.80	61.70	75.60
	Moderate	43.75	29.41	32.20	17.20	31.30	33.33
	Severe	75.00	59.09	64.00	50.00	68.50	56.30
Support vector machines	Normal	63.86	71.04	60.11	56.52	65.74	73.28
	Moderate	49.88	59.99	50.09	49.77	49.88	50.00
	Severe	71.78	81.48	59.94	64.63	72.49	81.48
Back propagation neural networking	Normal	50.00	47.77	32.85	40.00	42.38	42.22
	Moderate	29.04	23.33	6.67	2.22	7.14	1.12
	Severe	20.95	28.88	14.76	11.12	16.19	14.44
**Spaghetti meat**							
Support vector machines	Normal fillet without spaghetti	69.38	50.00	57.06	60.00	52.15	52.22
	Normal fillet with spaghetti	53.33	50.00	45.5	22.2	50.00	52.35
Back propagation neural networking	Normal fillet without spaghetti	100.00	52.95	65.51	42.30	58.62	50.00
	Normal fillet with spaghetti	100.00	75.00	29.31	19.23	29.31	11.50

*Woody breast*

a *n = 300 (normal = 148, moderate = 82, severe = 70); spaghetti meat*

b *n = 84; All data*

c *-WB scores, fillet weight, resistance, reactance, protein index, and fat index; without fillet weights*

d *-woody breast (WB) scores, resistance, reactance, protein index, and fat index; and Data without fat and protein index*

e *-WB scores, fillet weight, resistance, and reactance*.

The linear discriminant analysis technique is used to find a linear transformation that discriminates between various groups. However, LDA cannot find a lower-dimensional space if the groups are non-linearly separable. In other words, where discriminatory knowledge is not in the means of classes, LDA fails to locate the LDA space. One of the significant issues with the LDA methodology is singularity, also known as small sample size or under-sampling. This issue arises because of high-dimensional trend classification problems or a low number of training samples available for each class compared with the dimensionality of the sample space (Huang et al., 2002; Lu et al., 2005; Zhuang and Dai, 2005; Su et al., 2017; Tharwat et al., 2017).

The machine learning theory lays the groundwork for support vector machine, and this algorithm has gained widespread attention because of its unique performance efficiency, and ability to accomplish pinpoint accuracy and manage high-dimensional, multi-variate data sources. Cortes and Vapnik (1995) implemented SVMs as a new ML technique for two-group classification problems. Researchers have reported that SVMs are an economical, sensitive, and easy to use classifier that can be implemented in organised evaluation assignments (Vapnik, 1995). The inspection of large collected data sets during production is a significant application of SVM (Burbidge et al., 2001; Chinnam, 2002). SVM is frequently used in various food production environments, including product monitoring systems, mechanical fault detection, and dimensional accuracy (Ribeiro, 2005; Salahshoor et al., 2011; Çaydaş and Ekici, 2012; Azadeh et al., 2013). SVMs are used in different processing areas, such as drug designing and discovery, surgery, and cancer treatment, in addition to the food product processing industry (Vapnik, 2013). Product quality control (Borin et al., 2006), polymer recognition, and other applications are also possible (Li et al., 2009) areas in which SVM can be incorporated. These examples from different industries demonstrate that SVM algorithms have a broad range of applicability and versatility (Kotsiantis et al., 2007). This research demonstrates the ability for the implementation of SVM and BPNN in combination with BIA and fillet weight data to classify WB and SM fillets.

Statistical learning theory (SLT) is a robust and appropriate supervised learning algorithm for production research problems. Under SLT, algorithmic learning allows it to use an achieving function, representing the relationship between different components without being directly connected (Evgeniou et al., 2000). The algorithm enquires about the problem concerning how well the selected method resolves the problem, and accuracy prediction performance for previously unknown inputs is the subject of SLT (Evgeniou et al., 2000). A few more realistic techniques, such as autoencoders, SVM, and Bayesian optimization, are based on the theories of SLT (Battiti et al., 2002). SVM is considered a mathematical expression in its most basic form, a method (or algorithm) for optimising alphanumeric equations, with a given set of data (Noble, 2006).

The fundamental idea of support vector machine algorithmic expression can be easily understood by four fundamental concepts: (i) the imaginary hyper-plane, (ii) the margin of hyper-plane, (iii) the soft margin, and (iv) the kernel function (Tharwat, 2019). A solid line splits the region in half in two dimensions (Figure 4A), but we require a hypothetical plane to split the area into three dimensions. A hyper-plane is a collective term for a straight line in a high-dimensional region, and the dividing hyper-plane is the line that separates the pieces of data (Kecman, 2001; Tharwat, 2019). SVM, on the other hand, differs from other hyper-plane-based classifiers based on how the hyper-plane is chosen. Consider the grouping shown in Figure 4. By implementing SLT, it is easier to find the best possible plane to create the hyper-plane that will be used in the classification of data (Vapnik, 1963). The capability of SVM to classify the correct data points between given classes can be improved by using an imaginary hyper-plane in the space. The SLT theorem implies that the data used to train SVM originates from the same dataset as the data used to test it. For example, if an SVM algorithm is trained on the sensory property of a product, it cannot be used to train the data collected for the subjective response of consumers. Furthermore, we cannot expect SVM to work well if training is conducted with an SM breast fillet data set, so a WB data set is used for testing. At the same time, the SLT principle does not assume that two data sets come from the same class of distributions. For example, an SVM algorithm does not assume that training data values follow a normal distribution.

For a better understanding of support vector machine and its function, we have concluded an imaginary data set for classes A and B, which can be divided using a straight line. When the values in a data set are closer together or intersected (Figure 4B), SVM will manage this overlapping of data by inserting a soft margin. In essence, this causes specific data points to pass across the margin of the dividing hyper-plane, without influencing the outcome. The use of the soft margin provides the solution to the problem of misclassification (shown in Figure 4B) by considering the data point as an outlier (shown in Figure 4C). Another essential function for SVM classification is the kernel function (shown in Figures 4D,E), a mathematical trick that allows SVM to perform a two-dimensional classification of a one-dimensional data set. In general, a kernel function projects data from a low-dimensional space to a space of higher dimension.

Support vector machine classification efficiency (Table 4) for the separation of high dimensionality data showed better classification efficiency for normal (training efficiency 63.86%, testing efficiency 71.04%), moderate (training efficiency 49.88%, testing efficiency 59.99%), and severe WB (training efficiency 71.78%, testing efficiency 81.48%) compared with the LDA algorithm used by Morey et al. (2020). The BIA and fillet weight data set used in training the SVM performed well because of the higher dimensionality of the data set. When data are highly dimensional and the sample sets are relatively small, SVM analysis is more accurate to classify data, and has been used by other authors to help classify multi-dimensional data. Barbon et al. (2018) used a relatively small data set (*n* = 158) of NIR results combined with SVM (75::25) to classify normal and pale meat, as it relates to pale, soft, and exudative poultry breast meat. They demonstrated the use of SVM as a classification tool for breast fillets with muscle myopathies where the classification accuracy for normal fillets was 53.4 and 72. % for pale fillets. Geronimo et al. (2019), using an NIR system equipped with an image acquisition system, found 91.83% classification efficiencies (fillet images) using SVM to analyze a WB fillet sample set (sample size is unclear) with a 70::30 model. These researchers also used multilayer perceptron (a feed-forward network differing from the backpropagation network in BPNN) to classify the data set, and classification accuracy was 90.67% for WB. Yang et al. (2021) analysed images derived from the expressible fluid of breast meat to classify WB using SVM (training and testing ratio is unreported) and DL (training to testing is 2 to 1). These researchers found fewer classification efficiencies for SVM algorithms in the testing set (38.25–63.89%), compared with the training set (40.41–81.94%) in three out of the four SVM classification methods used. In their DL classification (a type of ANN) to classify WB, they reported 100% accuracy in the training set and 93.3% accuracy in the testing set.

Connexions of random different nodes or units in a computing system to solve problems that are impossible to solve by conventional statistical methods are known as artificial neural networks and are based on the circuitry of the human brain. When applied to a processor framework, the subconscious network can execute unique functions (perception, speech synthesis, image recognition), which have proven to be useful in industrial applications (Alpaydin, 2010). Neural networks allow an automated artificial skill to operate unsupervised reinforcement and classification algorithm functions (neural networks) by simulating the decentralised “data analysis” capabilities of the central nervous platform through neural networks (Pham and Afify, 2005; Corne et al., 2012). Decentralisation employs many necessary interconnected neurons or nodes and the capacity to process data through the complex response of these endpoints and their links to exogenous variables (Akay, 2011). These algorithms are crucial in the modern machine learning development of today (Nilsson, 2005) and can be classified into two categories: interpretation and algorithm. Neural networks are used in a variety of industrial sectors for a range of problems (Wang et al., 2005) e.g., process control, emphasising their key benefit and overall predictive validity (Pham and Afify, 2005). However, ANN (similar to SVM) requires a large sample size to attain maximum precision (Kotsiantis et al., 2007). Overfitting, which is linked to high-variance implementations, is universally acknowledged as a disadvantage of the ANN algorithm (Kotsiantis et al., 2007). Other difficulties with using neural networks include the sophistication of generated models, aversion for missing values, and, often, lengthy dataset training method (Pham and Afify, 2005; Kotsiantis et al., 2007).

For backpropagation neural networks, the data were pre-processed and consisted of just two dimensions with a lower level of classification complexity (Panchal et al., 2011). Classification efficiencies for the WB fillets using BPNN (Table 4) show that the testing data set for the normal (47.77%) and moderate fillets (23.33%) did not perform well, compared with the classification efficiency for the severe WB fillets (28.88%). The BPNN classification algorithm for the WB fillets did not perform well-because of the complexity of the data after pre-processing, and overfitting of the learning model due to the uneven distribution of weight on the input neuron layer. The BPNN classification algorithm for the SM data set (Table 4) performed well for the training data set for normal (training 100%, testing 52.95%) and SM fillets (training 100%, testing 75%). However, due to the complexity of pre-processed data, overfitting of BPNN and small data set, the classification efficiency of the testing set was lower than that of the training set. These studies all use SVM and ANN algorithms to classify small sample data sets, where the results always show that the accuracy in the training set data was higher than that in the testing set data, indicating that the training of the model is not performing well. Collection of larger data set for the supervised learning methods of classification provides the chances for getting lower error rates and better learning ability for the machine learning algorithms.

In a backpropagation neural network, the input data vector represents the pattern to be trained, and the output data vector represents the optimal set of output values that the network can generate when the training pattern is recalled. The aim of BPNN training is to reduce the total error between the expected and real outputs of the network (Panchal et al., 2011). To generate a reduction in error, the residual differences in the weights at each iteration must be unmeasurable. A learning rate metric, which reflects the rate of the move taken toward minimal error, must be defined to accomplish a reasonable training period. Learning will take too much time if this amount is too small, and if it is too high, the loss function will degenerate and errors will rise (Ganatra et al., 2011). When using neural networks to analyze WB data, overlearning or overfitting happens when the algorithm takes too long to run, and the network is too complicated for the problem or the amount of data available, whereas, to classify SM in a group of fillets, BPNN was used, and data are processed differently.

Additional analysis of the data was conducted to determine if the parameters used in data classification would make a difference in classification accuracies using the three methods (linear discriminant analysis, support vector machine, and backpropagation neural network) for both woody breast and spaghetti meat. Irrespective of the methodologies used, removing fillet weight reduced the classification accuracies of WB classification by up to 20%, indicating the importance of using fillet weights (for classification), which were significantly different among the WB categories (*p* < 0.05; Table 2). Removing the fat and protein indexes, which were not significantly different among the WB categories (*p* > 0.05; Table 3) increased the classification accuracy (Testing %) of the LDA models, while it was similar for the SVM and BPNN models. The finding indicates the significance of using fillet weight in the models and that the SVM and BPNN models analyze the significance of each parameter in the classification models.

It was interesting to note that the bioelectrical impedance analysis parameters, such as weight, were not significantly different between normal meat with and without spaghetti meat (Table 3). However, those parameters could be used collectively to develop classification models using LDA, SVM and BPNN models. Most importantly, removing weights did make a difference in the classification accuracies, but retaining the fillet weights and removing the fat and protein indexes did not necessarily increase the accuracy of the model. In the case of SM, more data are needed to build more accurate and stronger models.

## Conclusion

This project demonstrates the application of machine learning in poultry production processes to categorise chicken breast fillets into groups based on the severity of myopathy. The use of SVM and BPNN can be combined with BIA and fillet weight data to more accurately classify breast fillet myopathies, such as WB and SM, from normal breast fillets in real-time online, compared with the subjective hand palpation method. With the implementation of other meat quality parameters, such as water content, the classification accuracy of SVM and BPNN could be improved. To obtain a well-trained model for classification efficiency and to reduce overfitting and underfitting problems related to classification, future research should include larger data sets for breast fillet myopathies to avoid the overlapping of conditions caused by human error in the sorting of fillets. The innovative combination of these tools has the potential to improve poultry processing efficiencies and downgrades of breast fillets affected by undesirable myopathies while reducing customer complaints.

## Data Availability Statement

The raw data supporting the conclusions of this article will be made available by the authors, without undue reservation.

## Ethics Statement

Ethics approval was not required for this study in line with national/local guidelines.

## Author Contributions

AM is the lead investigator who conceptualised the idea, secured funding, and conducted the research. AM, AS, and JV conducted the bioelectrical impedance analysis data collection. AS performed the SVM, BPNN analysis, and manuscript preparation. AES and SS have helped in the cross-validation of analysis results and review of the manuscript. AM and LG assisted in writing—review and editing. All authors contributed to the article and approved the submitted version.

## Conflict of Interest

The authors declare that the research was conducted in the absence of any commercial or financial relationships that could be construed as a potential conflict of interest.

## Publisher's Note

All claims expressed in this article are solely those of the authors and do not necessarily represent those of their affiliated organizations, or those of the publisher, the editors and the reviewers. Any product that may be evaluated in this article, or claim that may be made by its manufacturer, is not guaranteed or endorsed by the publisher.
